# MRI-based cartilaginous acetabular coverage after open reduction for developmental dysplasia of the hip: association with hip function and a potential compensatory role in residual dysplasia

**DOI:** 10.3389/fped.2025.1731820

**Published:** 2025-12-18

**Authors:** Lijuan Wang, Guangbin Wang

**Affiliations:** 1 Shandong Provincial Hospital, Shandong University, Jinan, Shandong, China; 2 Department of Radiology, Yantai Yuhuangding Hospital, Qingdao University, Yantai, Shandong, China; 3 Department of Radiology, Shandong Provincial Hospital Affiliated to Shandong First Medical University, Jinan, Shandong, China

**Keywords:** acetabular coverage, children, DDH, developmental hip dysplasia, hip function, MRI, residual acetabular dysplasia

## Abstract

**Purpose:**

To determine the value of MRI-based cartilaginous acetabular coverage in assessing postoperative hip function in patients with DDH following open reduction surgery and to explore its potential compensatory role in cases of residual dysplasia.

**Methods:**

This retrospective study evaluated 78 hips from 59 children who had undergone open reduction for DDH. The postoperative clinical function of each hip was assessed using the modified MacKay criteria, and hips were dichotomized into a Good-Function group (Grades I and II; *n* = 40) and a Poor-Function group (Grades III and IV; *n* = 38). For all hips, radiographic (Acetabular Head Index, AHI) and MRI-based cartilaginous parameters (anterior and lateral Cartilaginous Acetabular-Head Index, A-CAHI and L-CAHI) were measured. Receiver Operating Characteristic (ROC) curve analysis was utilized to assess the diagnostic performance of these parameters in discriminating between the functional outcome groups. Furthermore, a subgroup analysis was performed on hips with Residual Acetabular Dysplasia (RAD; defined as Acetabular Index >20°) to compare imaging parameters based on functional status.

**Results:**

The diagnostic performance of MRI-based parameters A-CAHI and L-CAHI in discriminating between the two functional outcome groups was high, with Area Under the Curve (AUC) values of 0.893 and 0.881, respectively. A combined MRI model achieved the highest diagnostic performance (AUC = 0.918), significantly outperforming the radiographic AHI (AUC = 0.782; *p* = 0.002). In the subgroup analysis of hips with Residual Acetabular Dysplasia (RAD), those with satisfactory function exhibited significantly better cartilaginous coverage compared to the unsatisfactory group (*p* < 0.0001 for both A-CAHI and L-CAHI). Compared to non-dysplastic controls who also had good function, the RAD-Good subgroup demonstrated significantly inferior bony anatomy (e.g., mean AHI 71.88 vs. 80.42, *p* = 0.0030).

**Conclusion:**

MRI-based assessment of cartilaginous acetabular coverage is a superior tool for evaluating functional status after DDH surgery compared to traditional radiography. Our findings demonstrate that excellent cartilage development can compensate for underlying residual bony dysplasia, playing an important role in maintaining good hip function. Therefore, incorporating MRI into the clinical assessment of this challenging patient population is valuable for guiding clinical decision-making.

## 1 Introduction

Developmental dysplasia (DDH) is a chronic condition requiring long-term management. Although early interventions such as Pavlik harness application, bracing, and closed or open reduction can achieve concentric reduction, this does not guarantee complete normalization of the hip joint by skeletal maturity. Acetabular remodeling is a biological process spanning several years. A subset of patients may still present with residual hip dysplasia in adulthood, which is a primary risk factor for developing long-term osteoarthritis and requiring secondary osteotomy or even total hip arthroplasty. Compared with closed reduction, this challenge is particularly pronounced in the cohort of children requiring open reduction.

Sufficient acetabular coverage of the femoral head, incorporating both osseous and cartilaginous components, ensures hip joint stability and physiological biomechanical stimulation. These factors serve as fundamental prerequisites for proper acetabular remodeling and ultimately determine long-term hip function (1).

Conventional radiography remains a widely employed modality for evaluating reduction quality (2–6). However, due to the abundant cartilaginous composition of the pediatric hip joint, radiographs can only depict osseous structures, often resulting in an underestimation of both the actual femoral head coverage and the intrinsic potential for acetabular remodeling. This may lead to unwarranted surgical interventions (2, 4). Furthermore, given that dysplastic hips can exhibit deficiencies in various locations, including anteriorly, posteriorly, or globally, as described by Nepple and Wilkin et al. (7, 8), radiographs, as two-dimensional imaging techniques, fail to capture the full spectrum of the deformity (9). While three-dimensional Computed Tomography (CT) is the gold standard for bone characterization, its radiation exposure and inability to visualize cartilage limit its routine use in children (3, 10).

Magnetic Resonance Imaging (MRI) can clearly outline the anatomical structure of cartilage and enable multiplanar imaging, overcoming these limitations of radiography. The cartilaginous acetabulum is fully developed at birth and extends superiorly to cover the femoral head in a configuration morphologically comparable to that of the mature osseous acetabulum. Accumulating evidence suggests that the cartilaginous anlage of the acetabulum in pediatric patients with DDH may serve as a more accurate predictor of ultimate acetabular morphology (11–16).

While previous studies have utilized MRI to investigate parameters such as the cartilaginous acetabular index (CAI) and cartilaginous acetabular head index (CAHI) to assess acetabular morphology, femoral head coverage, and predict acetabular remodeling, these researches have predominantly relied on coronal plane imaging to evaluate the lateral acetabular morphology and coverage (17–20). Only a few studies have incorporated the axial plane to assess anterior coverage (13, 21), and there is a significant paucity of research evaluating anterior cartilaginous acetabular coverage on the oblique sagittal plane. As is known to all, the hip is a ball-and-socket joint, and in addition to the most studied lateral coverage, a large proportion of DDH patients exhibit insufficient anterior coverage due to acetabular anteversion, which similarly compromises hip joint stability and function (22). Therefore, a more comprehensive, three-dimensional assessment should incorporate this equally crucial anterior cartilaginous structural parameter.

In addition, the majority of prior MRI studies have shared a common design paradigm: using early postoperative imaging to predict future endpoints, such as the eventual development of residual acetabular dysplasia (RAD) or the need for secondary surgery (13, 17, 23–25). Consequently, this “predicting the future” model does not directly address a more fundamental question faced by clinicians during the critical mid-term decision-making period: what is the anatomical basis for this child's current functional status?

This also leads to another noteworthy clinical question. Clinically, a subset of DDH patients present with radiographic evidence of RAD, yet paradoxically maintain good hip function, which complicates secondary surgical decision-making. We hypothesize that in these patients, superior cartilaginous development, encompassing both lateral and anterior aspects, may compensate for the morphological osseous deficiency, thereby preserving joint stability and function.

Therefore, the aim of this study was to establish a direct correlation between multi-planar MRI–based cartilaginous acetabular coverage at mid-term follow-up and the contemporaneous clinical function of pediatric patients after open reduction for developmental dysplasia of the hip (DDH), to determine the value of MRI-based cartilaginous coverage in assessing postoperative hip function, and to explore its potential compensatory role in cases of residual dysplasia. To achieve this aim, the study focused on the following specific objectives:

To compare multi-planar MRI-derived cartilaginous coverage parameters with traditional radiographic measures in differentiating patients with good vs. poor functional outcomes.
To evaluate whether enhanced cartilaginous coverage can compensate for residual osseous dysplasia in patients who present with radiographic RAD but maintain good clinical function.

## 2 Materials and methods

### 2.1 Study participants

Ethical approval for this retrospective study, including a waiver of informed consent, was granted by the Biomedical Research Ethics Committee Involving Humans of Shandong Provincial Hospital (Approval No. SWYX: 2025-650).

We reviewed the imaging data of patients with DDH who underwent imaging examinations at our institution between 2019 and 2022. Inclusion criteria were as follows: (1) Patients who had previously undergone open reduction (including those who had received closed reduction or other conservative treatments prior to the final open reduction), were under 8 years old, and had an unclosed Y-shaped cartilage; (2) MRI and x-ray examinations performed within a one-week interval, with both conducted in the 4–6-year postoperative period after open reduction; (3) Femoral head and acetabulum were well matched confirmed on the follow-up MRI (On the coronal and axial views passing through the triradiate cartilage of the acetabulum, the femoral head is roughly located in the center of the acetabulum); and (4) complete medical records. This specific cohort and follow-up period were selected because they represent a clinically challenging population at high risk for RAD (26). This mid-term window is a critical decision-making point where the trajectory of acetabular remodeling has become apparent, yet significant growth potential remains, making the assessment for potential secondary surgery particularly crucial (27). Patients with teratologic hip dislocation, hip subluxation, femoral deformities (including femoral head deformity, avascular necrosis of the femoral head, and coxa plana), Perthes’ disease, cerebral palsy, metabolic diseases (such as mucopolysaccharidosis or mucolipidosis), a history of other surgical interventions (such as pelvic or femoral osteotomies), or incomplete data were excluded. A total of 78 hips from 59 patients met the inclusion criteria and were included in the final analysis.

Clinical outcomes were assessed using the modified McKay criteria (Table 1). According to the functional scores, the hips were subsequently divided into two groups for analysis: the Good-Function group (Grades I and II) and the Poor-Function group (Grades III and IV).

**Table 1:** Mckay's criteria for clinical evaluation of developmental dysplasia of the Hip (DDH).

Grade	Rating	Description
I	Excellent	Stable, painless hip; no limp; negative Trendelenburg's sign; full range of motion
II	Good	Stable, painless hip; slight limp or slight decrease in range of motion; negative Trendelenburg's sign
III	Fair	Stable hip; minimal or no pain; moderate limp or moderate loss of motion; positive Trendelenburg's sign
IV	Poor	Unstable hip; hip pain; marked limp and/or stiffness

Clinical grading system for outcomes after treatment of developmental dysplasia of the hip, as described by McKay. Grades are based on hip stability, presence of pain, gait abnormalities, Trendelenburg's sign, and range of motion.

### 2.2 Imaging parameter measurement

All radiological and MRI parameters were measured by two experienced radiologists (Observer 1 and Observer 2), who had received training on the measurement methods of each parameter prior to the measurement and were blinded to each other's measurement results as well as the patients' clinical data. To assess inter- and intra-rater reliability, 40 hips were randomly selected from the cohort.

For intra-rater reliability, Observer 1 re-measured the parameters for these 40 hips after a four-week interval. For inter-rater reliability, Observer 2 independently measured the same set of 40 hips. The reliability was evaluated using the intraclass correlation coefficient (ICC). Interpretation of ICC values was based on the guidelines by Koo and Li (28), where values between 0.75 and 0.90 indicate good reliability and values greater than 0.90 indicate excellent reliability. As the reliability was confirmed to be high, the data collected by Observer 1 in the first measurement session were used for the final statistical analysis.

### 2.3 MRI protocol and definition of imaging parameters

All MRI examinations were performed on a 3.0 T scanner (Magnetom Skyra, Siemens Healthineers, Erlangen, Germany) using an 18-channel phased-array body coil. Patients were positioned supine with both hips in neutral extension and the pelvis aligned symmetrically. The feet were maintained in 15°–20° of internal rotation to ensure anterior orientation of the patellae. Younger children unable to cooperate were sedated with oral chloral hydrate (50 mg/kg) approximately 30 min prior to the examination.

Imaging was performed in accordance with a specialized multidisciplinary protocol developed jointly by our Pediatric Orthopedics and Radiology departments. To ensure a comprehensive assessment, this protocol integrates standard acquisition planes with customized institutional sequences optimized for DDH evaluation:

Standard Protocol: Consistent with established guidelines for post-reduction assessment (29), the routine acquisition included Coronal T1-weighted imaging (T1WI) to evaluate osseous morphology, and Axial/Coronal Fat-Suppressed T2-weighted imaging (FS-T2WI) to assess joint effusion and congruency.
Specialized Institutional Sequence: To specifically address the limitation of standard views in assessing anterior coverage, a high-resolution Oblique Sagittal Fat-Suppressed Proton Density Weighted Imaging (FS-PDWI) sequence was included. This sequence, developed through multidisciplinary collaboration between our orthopedic and radiology departments, is routinely utilized to optimize the visualization of the anterior acetabular cartilage and labrum (29).

For the specific objectives of this study, quantitative measurements were derived from the Coronal T1WI (for lateral cartilaginous coverage) and the specialized Oblique Sagittal FS-PDWI (for anterior cartilaginous coverage).

**Figure 1:**
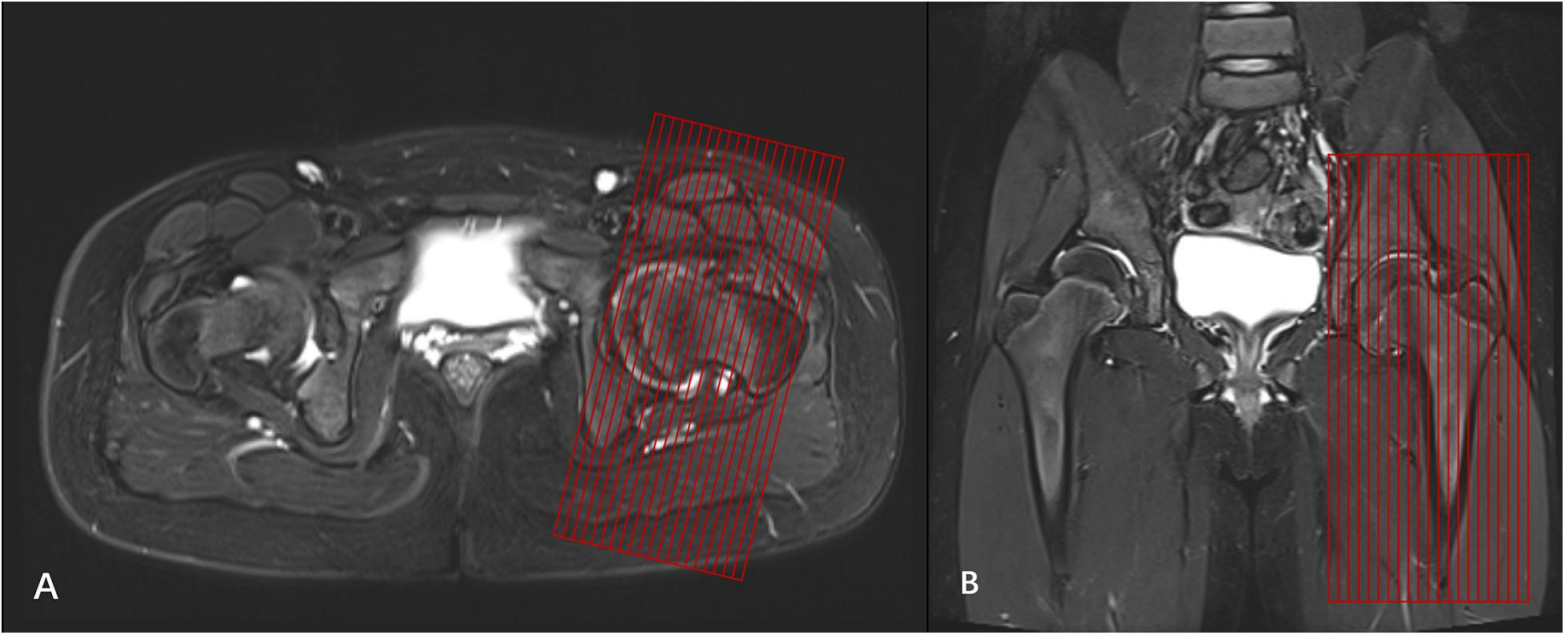
Method for positioning high-resolution oblique sagittal scanning of the hip joint. **(A)** On the transverse image, the scanning centerline is perpendicular to the femoral neck. **(B)** On the coronal image, the scanning line is parallel to the long axis of the femur.

The imaging protocol commenced with conventional multiplanar scans of both hips for localization. Subsequently, a high-resolution oblique sagittal acquisition of the affected hip was performed. The oblique sagittal plane was planned from axial images by aligning the scan slices perpendicular to the long axis of the femoral neck. This alignment was then refined on the coronal images to be parallel to the long axis of the femoral shaft (Figures 1A,B).

The high-resolution acquisition utilized an oblique sagittal fat-suppressed proton density-weighted imaging (FS-PDWI) sequence with the following parameters: Repetition Time (TR)/Echo Time (TE), 2,000/24 ms; Field of View (FOV), 160 × 160 mm; slice thickness, 3.5 mm; intersection gap, 0.35 mm; matrix, 256 × 320; number of signal averages (NSA) or excitations (NEX), 2.

**Figure 2:**
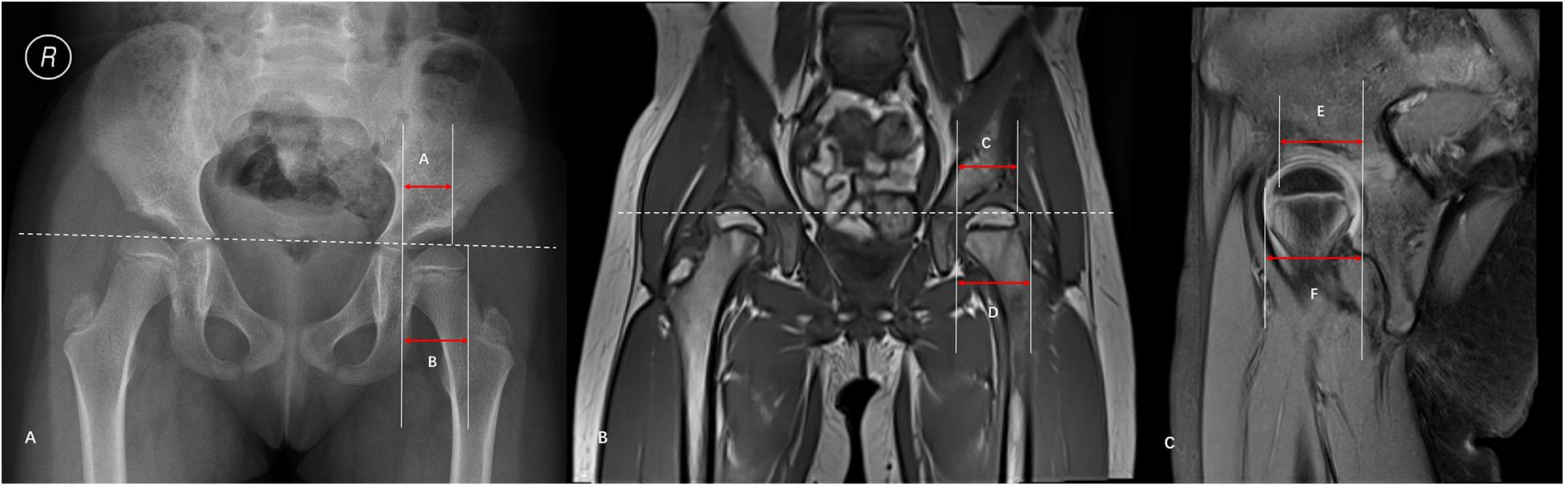
Measurement of the osseous and cartilaginous acetabular head indices in a 5-year-old girl with left developmental dysplasia of the hip (DDH) and grade II hip function at the time of imaging. The patient had undergone an open reduction of the left hip at 1.5 years of age. The images **(A–C)** demonstrate the measurement methods, yielding an osseous Acetabular Head Index (AHI) of 74.6%, a Lateral Cartilaginous AHI (L-CAHI) of 81.2%, and an Anterior Cartilaginous AHI (A-CAHI) of 87.1%. **(A)** Anteroposterior (AP) radiograph. A Is the distance from the medial edge of the femoral head to the lateral edge of the acetabular sourcil, and B is the maximum diameter of the femoral head. AHI = A/B × 100%. **(B)** Coronal T1-weighted image (T1WI). C Is the distance from the most medial edge of the femoral head cartilage to the most lateral edge of the acetabular cartilage, and D is the maximum diameter of the femoral head. L-CAHI = C/D × 100%. **(C)** Oblique sagittal proton density-weighted image with fat suppression. E is the distance from the most posterior edge of the femoral head cartilage to the most anterior edge of the acetabular cartilage, and F is the maximum anteroposterior diameter of the femoral head A-CAHI = E/F × 100%.

The following parameters were measured:

Radiographic Evaluation: On pelvic anteroposterior (AP) radiographs, the Acetabular Head Index (AHI) was measured to assess lateral femoral head coverage, according to the standard method described by Heyman and Herndon (30). The AHI was calculated by measuring the distance from the medial-most edge of the osseous femoral head to the lateral-most edge of the osseous acetabulum (A) and the maximum transverse diameter of the osseous femoral head (B). The index was calculated using the formula: AHI = (A/B) × 100% (Figure 2A).
MRI Evaluation of Lateral Coverage: To assess the true anatomical coverage including the cartilaginous acetabulum, the lateral cartilaginous acetabulum-head index (L-CAHI) was utilized based on the method described by Douira-Khomsi et al. (31). On coronal T1-weighted images (T1WI), the measurement was performed on the slice depicting the maximum diameter of the femoral head. The distance from the medial-most edge of the femoral head cartilage to the lateral-most edge of the acetabular cartilage was measured as (C), along with the maximum transverse diameter of the femoral head cartilage (D). The L-CAHI was calculated as: L−CAHI = (C/D) × 100% (Figure 2B).

MRI Evaluation of Anterior Coverage: To evaluate anterior cartilaginous coverage, the anterior cartilaginous acetabulum-head index (A-CAHI) was measured on oblique sagittal fat-suppressed proton density-weighted images (FS-PDWI), following the same measurement principles described by Douira-Khomsi et al. (31). On the slice showing the maximum anteroposterior diameter of the femoral head, the distance from the posterior-most edge of the femoral head cartilage to the anterior-most edge of the acetabular cartilage was measured as (E), and the maximum anteroposterior diameter of the femoral head cartilage was measured as (F). The A-CAHI was calculated with the formula: A−CAHI = (E/F) × 100% (Figure 2C).

### 2.4 Subgroup definition

To further investigate the interplay between osseous morphology and cartilaginous development on functional outcomes, patients were stratified into three distinct subgroups:

**Group A (RAD-Good):** Patients with RAD but good functional outcome (X_AI > 20° and Hip Functio*n* = good).
**Group B (RAD-Poor):** Patients with RAD and poor functional outcome (X_AI > 20° and Hip Function = poor).
**Group C (Non-RAD-Good):** Patients without RAD and with good functional outcome (X_AI <= 20° and Hip Function = good).

### 2.5 Sample size justification

Since this was a retrospective study, the sample size was strictly determined by the inclusion criteria. To verify the statistical sufficiency of the included cohort (*n* = 78), a *post-hoc* power analysis was performed using G*Power software (32). Based on the primary outcome (A-CAHI), the observed data yielded a large effect size (Cohen's *d* = 1.71). With a two-sided α level of 0.05, the calculated statistical power (1—ß) exceeded 0.99, confirming that the sample size was adequate to detect significant differences between the functional groups*.*

### 2.6 Statistical analysis

All statistical analyses were performed using Python (version 3.x) with the scipy, pandas, and scikit-learn libraries. Continuous variables were presented as mean ± standard deviation (SD) or median and interquartile range (IQR) based on data distribution. Categorical variables were presented as frequencies and percentages.

Differences between the Good and Poor function groups were assessed using the independent *t*-test or Mann–Whitney *U* test for continuous variables and the Chi-square test or Fisher's exact test for categorical variables. The Mann–Whitney *U* test was used for subgroup comparisons. A *p*-value < 0.05 was considered statistically significant.

Receiver Operating Characteristic (ROC) curve analysis was conducted to evaluate the performance of A-CAHI, L-CAHI, and AHI in discriminating between poor and good hip function at the time of assessment. A combined predictive model incorporating both A-CAHI and L-CAHI was also developed using binary logistic regression, and its performance was similarly evaluated. The Area Under the Curve (AUC), optimal cut-off value (determined by the Youden index), sensitivity, and specificity were calculated for each model. The DeLong test (33) was used to assess for statistically significant differences between the AUCs of correlated ROC curves.

## 3 Results

### 3.1 Patient demographics and baseline characteristics

The demographic and clinical characteristics of the 78 hips, categorized by hip function group, are presented in Table 2. No significant differences were found between the Good-Function group and the Poor-Function group (*n* = 38) groups regarding the mean age at reduction (*p* = 0.235), the prevalence of bilateral DDH (*p* = 0.505), or laterality (*p* = 0.218). A statistically significant difference was noted in sex distribution, with a higher proportion of females in the Good-Function group (100.00% vs. 86.84%, *p* = 0.024).

**Table 2:** Comparison of demographic and clinical characteristics between groups.

Characteristic	Total Cohort (*N* = 78)	Good Function group (*n* = 40)	Poor Function group (*n* = 38)	*p*-value (Hip Function 1 vs. 2)
Reduction Age	1.38 ± 0.61	1.46 ± 0.66	1.29 ± 0.54	0.235
No. (%) famale	73 (93.6)	40 (100.00%)	33 (86.84%)	0.024
Bilateral DDH	38 (48.7)	18 (45.00%)	20 (52.63%)	0.505
No. (%) of left hips	27 (34.6)	14 (35.00%)	13 (34.21%)	0.218
No. (%) of right hips	13 (16.7)	8 (20.00%)	5 (13.16%)	0.218
L-CAHI	75.78 ± 7.17	80.14 ± 5.28	71.19 ± 5.94	<0.001
A-CAHI	78.31 ± 9.59	84.39 ± 6.19	71.91 ± 8.31	<0.001
AI	23.36 ± 7.17	21.10 ± 6.55	25.74 ± 7.11	0.002
AHI	71.10 ± 12.65	76.58 ± 9.66	65.34 ± 12.98	<0.001

Data are presented as mean ± standard deviation or number (percentage). *p*-values are for comparisons between the good function group and the poor function group. DDH, developmental dysplasia of the hip; L-CAHI, lateral cartilaginous acetabular head index; A-CAHI, anterior cartilaginous acetabular head index; AI, acetabular index; AHI, acetabular head index.

### 3.2 Comparison of radiological and MRI parameters

All radiological parameters measured showed significant differences between the two functional outcome groups. Compared to the Poor-Function group, the Good-Function group exhibited significantly higher values for L-CAHI (*p* < 0.001), A-CAHI (*p* < 0.001), and AHI (*p* < 0.001). Conversely, the AI was significantly lower in the Good-Function group (*p* = 0.002) (Table 2).

### 3.3 Diagnostic performance of imaging parameters for concurrent hip function

**Figure 3:**
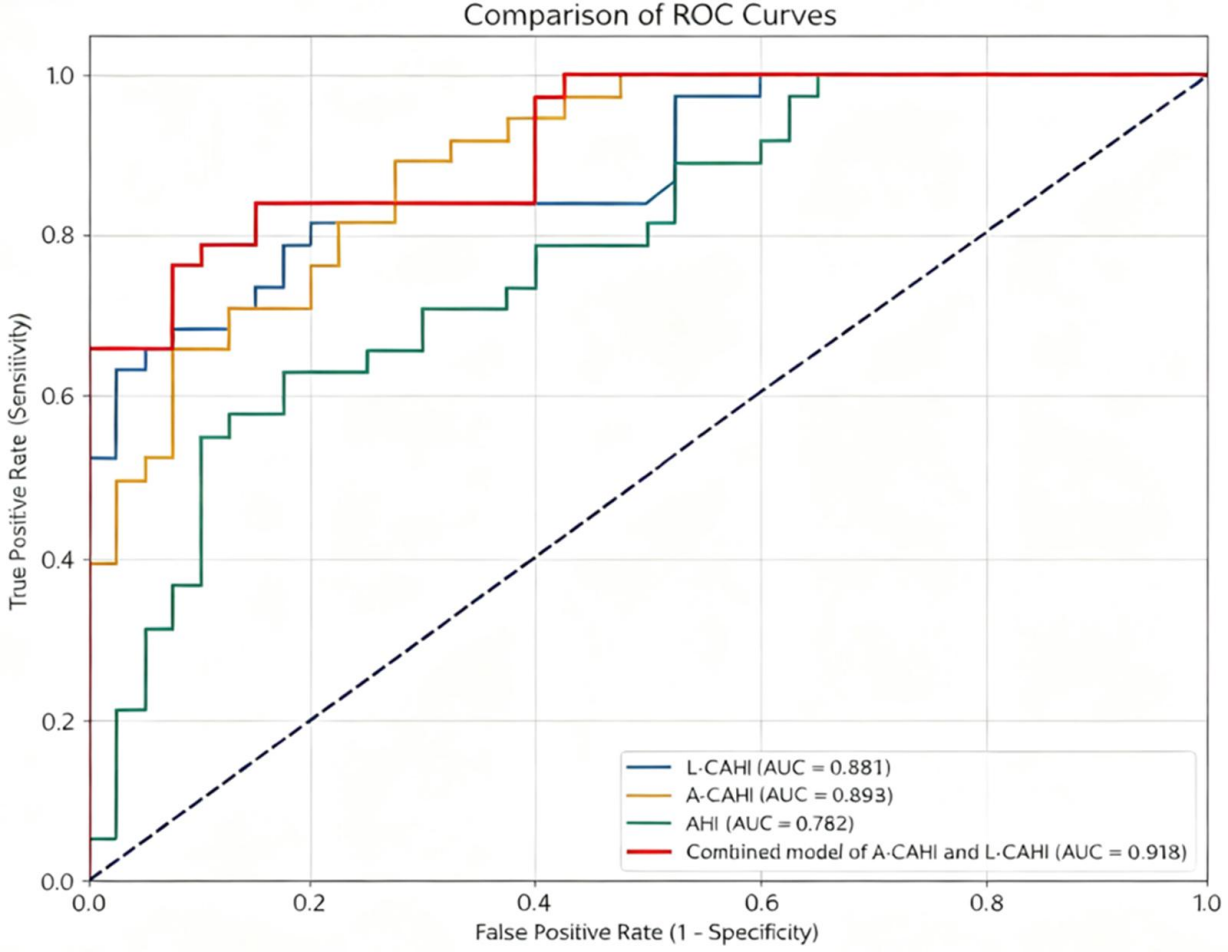
Comparison of receiver operating characteristic (ROC) curves for different imaging parameters and the combined model in discriminating between good and poor hip function. The graph depicts the diagnostic performance of MR_L-CAHI (area under the curve, AUC = 0.881), MR_A-CAHI (AUC = 0.893), X_AHI (AUC = 0.782), and a model combining MR parameters (AUC = 0.918). The combined model demonstrates superior predictive ability with the highest AUC value.

The results of the ROC curve analysis for discriminating poor from good hip function are summarized in Table 3. The combined model of A-CAHI and L-CAHI yielded the highest AUC of 0.918, followed by A-CAHI (AUC = 0.893) and L-CAHI (AUC = 0.881). The radiographic parameter AHI demonstrated moderate diagnostic value with an AUC of 0.782. The ROC curves for all Parameters are presented in Figure 3.

**Table 3:** ROC analysis of imaging parameters for predicting poor functional outcome.

Parameter	AUC	Cutoff	Sensitivity	Specificity
A-CAHI	0.893	79.71	89.50%	72.50%
L-CAHI	0.881	75.73	81.60%	80.00%
AHI	0.782	70.31	63.20%	82.50%
Combined Model (A + L-CAHI)	0.918	/	84.20%	85%

A-CAHI, anterior cartilaginous acetabular head index; L-CAHI, lateral cartilaginous acetabular head index; AHI, acetabular head index; Combined Model (A + L-CAHI), combined model of A-CAHI and L-CAHI.

### 3.4 Comparison of ROC curves

Pairwise comparison of the AUCs using the DeLong test revealed that the combined model of A-CAHI and L-CAHI had a significantly higher diagnostic performance than the conventional AHI (ΔAUC = 0.136, *p* = 0.021), as detailed in Table 4. Furthermore, although not reaching statistical significance, both L-CAHI (ΔAUC = 0.099, *p* = 0.116) and A-CAHI (ΔAUC = 0.111, *p* = 0.069) showed a trend toward higher AUC values compared to AHI. Aside from the comparisons mentioned, no other pairwise comparisons among the indices or between the combined model and the individual indices reached statistical significance.

**Table 4:** Delong test for pair-wise comparison of areas under the ROC curve (AUC).

Comparison^a^	AUC (95% CI)	AUC (95% CI)	ΔAUC	SE ΔAUC^b^	*z*-value	*p*-value^c^
L-CAHI vs. A-CAHI	0.881 (0.807–0.954)	0.893 (0.827–0.960)	0.012	0.051	0.24	0.81
L-CAHI vs. AHI	0.881 (0.807–0.954)	0.782 (0.681–0.883)	0.099	0.063	1.57	0.116
A-CAHI vs. AHI	0.893 (0.827–0.960)	0.782 (0.681–0.883)	0.111	0.061	1.82	0.069
Combined model^d^ vs. L-CAHI	0.918 (0.860–0.976)	0.881 (0.807–0.954)	0.037	0.048	0.77	0.441
Combined model^d^ vs. A-CAHI	0.918 (0.860–0.976)	0.893 (0.827–0.960)	0.025	0.045	0.56	0.576
Combined model^d^ vs. AHI	0.918 (0.860–0.976)	0.782 (0.681–0.883)	0.136	0.059	2.31	0.021

a DeLong et al. method used for all comparisons.

b Standard error of the difference between the two AUCs.

c Two-tailed; unadjusted. Bonferroni-corrected threshold for six simultaneous comparisons: 0.0083.

d Combined model = logistic combination of L-CAHI and A-CAHI.

### 3.5 Subgroup analysis

The cohort was stratified into Group A (RAD-Good, *n* = 18), Group B (RAD-Poor, *n* = 30), and Group C (Non-RAD-Good, *n* = 22) (Table 5).

#### 3.5.1 Comparison of patients with residual dysplasia (group A vs. group B)

Among patients with residual acetabular dysplasia (AI > 20°), those with good function (Group A) had significantly better cartilaginous and osseous parameters than those with poor function (Group B). The median A-CAHI (80.77 vs. 72.23, *p* < 0.001), L-CAHI (76.59 vs. 70.93, *p* < 0.001), and AHI (73.06 vs. 66.55, *p* = 0.002) were all significantly higher in Group A (Table 5).

**Table 5:** Comparison of imaging parameters between group A and group B.

Parameter	Group A (RAD-Good, *n* = 18)	Group B (RAD-Poor, *n* = 30)	Group C (Non-RAD-Good, *n* = 22)	*p*-value (A vs. B)	*p*-value (A vs. C)
AHI	73.06 [70.95–73.97]	66.55 [59.32–71.01]	80.61 [73.86–87.40]	0.002	0.005
L-CAHI	80.77 [78.91–85.46]	72.23 [65.96–75.92]	/	<0.001	/
A-CAHI	76.59 [74.98–80.29]	70.93 [67.27–73.51]	/	<0.001	/

Data are presented as median [interquartile range]. Group A (patients with RAD but good functional outcome, X_AI > 20°, *n* = 18) showed significantly higher AHI, L-CAHI, and A-CAHI values compared to group B (Patients with RAD and poor functional outcome, X_AI > 20°, *n* = 30). Group A also had a significantly lower AHI compared to group C (patients without RAD and with good functional outcome, X_AI <= 20°, *n* = 22). RAD, residual acetabular dysplasia; AHI, acetabular head index; L-CAHI, lateral cartilaginous acetabular head index; A-CAHI, anterior cartilaginous acetabular head index; *P*-values were calculated using the Mann–Whitney *U* test.

#### 3.5.2 Comparison of patients with good function (group A vs. group C)

To validate the underlying osseous deficiency in Group A, their bone structure was compared to that of patients with good outcomes and no residual dysplasia (Group C). As hypothesized, the AHI was significantly lower (worse) in Group A compared to Group C (median 73.06 vs. 80.61, *p* = 0.005), confirming that the RAD-Good group indeed had a poorer osseous foundation despite their favorable clinical outcome (Table 5).

## 4 Reliability of measurements

The intra- and interobserver reliability of the measurements were assessed using intraclass correlation coefficients (ICCs), and the results are presented in Table 6.

**Table 6:** Assessment of intra- and interobserver agreement of acetabular head index, lateral cartilaginous acetabular head index and anterior cartilaginous acetabular head index (ICC, intraclass correlation coefficient; CI, confidence interval).

Observers	Acetabular head index		Lateral cartilaginous acetabular head index		Anterior cartilaginous acetabular head index	
	ICC (95%CI)	*p*-value	ICC (95%CI)	*p*-value	ICC (95%CI)	*p*-value
Observer 1–1	0.977 [0.953, 0.989]	<0.001	0.933 [0.867, 0.967]	<0.001	0.961 [0.921, 0.981]	<0.001
Observer 1–2	0.955 [0.909, 0.978]	<0.001	0.881 [0.769, 0.941]	<0.001	0.890 [0.784, 0.945]	<0.001

Agreement is expressed as the intraclass correlation coefficient (ICC) with its 95% confidence interval (CI). All *p*-values were <0.001.

The intraobserver agreement was excellent for all three measurements. The ICC for the acetabular head index was 0.977 (95% CI, 0.953–0.989; *p* < 0.001). The lateral cartilaginous acetabular head index showed an ICC of 0.933 (95% CI, 0.867–0.967; *p* < 0.001), and the anterior cartilaginous acetabular head index had an ICC of 0.961 (95% CI, 0.921–0.981; *p* < 0.001).

The interobserver agreement was good to excellent. The ICC for the acetabular head index was 0.955 (95% CI, 0.909–0.978; *p* < 0.001), indicating excellent reliability. The lateral cartilaginous acetabular head index had an ICC of 0.881 (95% CI, 0.769–0.941; *p* < 0.001), and the anterior cartilaginous acetabular head index had an ICC of 0.890 (95% CI, 0.784–0.945; *p* < 0.001), demonstrating good reliability.

## 5 Discussion

Given the well-established limitations of radiography in assessing the cartilaginous-predominant pediatric hip, magnetic resonance imaging is increasingly being utilized to evaluate the quality of reduction. The most classic assessment metrics, such as the CAI and the cartilaginous center-edge angle (C-CEA), are primarily based on coronal plane measurements and thus describe lateral cartilaginous coverage (23, 34). However, DDH is a three-dimensional deformity, often accompanied by increased acetabular anteversion leading to deficient anterior coverage. Relying solely on lateral coverage may overestimate or underestimate the overall acetabular containment. Consequently, recent research has shifted its focus toward multi-planar assessment of acetabular coverage.

A study by Tsukagoshi et al. on the relationship between cartilaginous morphology and subsequent bony acetabular growth in two-year-olds after DDH reduction suggested that both lateral and anterior morphology were associated with future bony development, with anterior coverage (represented by an axial-based anterior C-CEA) being particularly crucial (13). A methodological limitation of the C-CEA measurement is its susceptibility to error when the femoral head is aspherical, as the center of the head is difficult to identify reliably (35). Furthermore, the measured value of CCEA may vary due to different positions of the hip joint and lower limbs (23).

The AHI, originally described by Heyman and Herndon (30), was modified for application to the cartilaginous infant hip to quantify femoral head containment. Douira-Khomsi et al. measured the cartilaginous AHI on both coronal (L-CAHI) and sagittal (A-CAHI) MRI views in 27 children with DDH, proposing preliminary thresholds of 85% for L-CAHI and 95% for A-CAHI as indicators of good cartilaginous coverage (31). However, these values were derived from internal observations within a small cohort, and the treatments received by subjects prior to MRI were inconsistent, necessitating further validation to establish normative data. Similar to the study by Douira-Khomsi et al., our study assessed both lateral and anterior cartilaginous acetabular head coverage (L-CAHI and A-CAHI) on coronal and oblique sagittal MR images. However, we enrolled a larger cohort and restricted the study population to children at mid-term follow-up after open reduction to minimize the potential confounding effects of differential treatment approaches. We then compared these MR-based cartilaginous indices with the osseous AHI from plain radiographs. Furthermore, unlike most prior studies that focused on predicting future acetabular development or the need for subsequent surgery, the present study sought to establish a direct correlation between concurrent cartilaginous coverage and the functional status of the hip, thereby providing an imaging-based rationale for secondary surgery decisions during this critical follow-up period. In this study, the intra- and interobserver reliability for all measurements was good to excellent, with ICCs ranging from 0.881 to 0.977, ensuring the consistency of our data. Demographically, our cohort exhibited a significant female predominance. This distribution aligns with large-scale epidemiological data recently reported by Jacobsen et al. (36), which reaffirmed female sex as a persistent and significant risk factor for DDH.

The principal finding of our study is that cartilaginous coverage is a superior predictor of concurrent hip function compared to osseous metrics. This was confirmed by ROC analysis, which showed that the AUCs for both A-CAHI (0.893) and L-CAHI (0.881) were significantly higher than that for the bony X_AHI (0.782). The high diagnostic performance of A-CAHI (AUC = 0.893, sensitivity = 89.50%, specificity = 72.50%) underscores the clinical importance of assessing anterosuperior coverage—a common site of deficiency in DDH that is undetectable by coronal-only assessment (22). Nakamura et al (21) utilized MRI to evaluate the three-dimensional acetabular morphology in children with DDH and healthy controls. Their main parameter, the femoral head coverage ratio (FHCR), quantifies cartilaginous acetabular coverage over the femoral head by calculating the ratio of acetabular depth to femoral head diameter. This principle is methodologically aligned with the CAHI used in our study. However, their measurement protocol involves more cumbersome procedures, requiring dedicated three-dimensional processing software and higher-resolution imaging sequences. They found that, compared with the healthy control group, children with DDH on the affected side before osteotomy had overall insufficient coverage of the cartilaginous acetabulum, which is similar to the results of our study: in our cohort, the anterior and lateral cartilage coverage rates in the poor-function group were also significantly lower than those in the good-function group (both *p*-values < 0.001).

Our study identified that an A-CAHI <79.71% and an L-CAHI <75.73% were indicative of insufficient anterior and lateral cartilaginous coverage, respectively, and associated with a higher probability of poor hip function. Notably, our L-CAHI threshold of 75.73% is comparable to the thresholds for insufficient lateral coverage (L-CAHI <77% and <73%) determined from the unaffected hips in two studies on Legg-Calvé-Perthes disease (37, 38).

In our study, a combined model incorporating both anterior and lateral parameters achieved the highest predictive efficacy (AUC = 0.918), confirming that a comprehensive, multi-directional assessment of the cartilaginous acetabulum is not only biomechanically sound but also holds superior clinical value in correlating anatomy with function.

Our subgroup analysis of patients with radiographic RAD reveals a clinically significant finding that provides quantitative evidence for the “cartilage compensation” hypothesis. We demonstrated that despite having an objectively poorer osseous foundation than the non-RAD control group, the RAD-Good group had significantly better cartilaginous coverage (median A-CAHI 80.77 vs. 72.23, *p* < 0.001) than the RAD-Poor group. This may help to explain the clinical-radiographic paradox (39), where some children with concerning radiographic findings maintain good hip function. It clinically validates the concept of “divergent dysplasia” described by Walbron et al., where sufficient cartilage coverage might compensate for underlying bony defects and portends a relative favorable prognosis (23, 24). Several studies have also reached similar conclusions. When predicting the future development of the acetabulum (23, 31), in addition to the evaluation of osseous structures, the assessment of the cartilaginous acetabulum is equally important. This is supported by Collins et al. (40), who noted that muscle and joint contact forces influence good hip joint containment. Since these forces are primarily transmitted through the cartilaginous interface in children, maintaining this containment allows the acetabulum to adapt and deepen. If the cartilaginous acetabulum is sufficient in the early stage, the coverage of the osseous acetabulum may grow adequately in the later stage, thereby avoiding premature secondary surgery (16). In A. Bos's study, 11 children with radiographic acetabular dysplasia exhibited adequate cartilaginous acetabular coverage on magnetic resonance imaging, obviating the need for acetabular surgery. Similarly, Wiem et al. documented that, among 31 hips with residual acetabular dysplasia, 27 demonstrated sufficient cartilaginous coverage in both the coronal and sagittal planes on MRI (mean cartilaginous coronal head index, 85%; mean cartilaginous sagittal head index, 95%), and all subsequently underwent spontaneous progressive ossification. These observations collectively corroborate the foregoing concept (4, 31).

These findings suggest potential clinical implications. For patients with RAD, an A-CAHI ≥79.71 or an L-CAHI ≥75.73 suggests a high likelihood of good hip function, potentially justifying the continuation of conservative management even when radiographic findings appear unfavorable. Figure 2 shows the radiographic and MR images of a 5-year-old girl with RAD who previously underwent open reduction for left DDH at 1.5 years of age. Although radiographs demonstrated poor osseous coverage consistent with RAD (AI = 23.4°, AHI = 74.6%), MRI revealed good cartilaginous coverage (L-CAHI = 81.2%, A-CAHI = 87.1%). Functional follow-up according to the modified McKay criteria was excellent (Grade II). Therefore, continued conservative observation was recommended over immediate acetabular osteotomy. The combined MRI model can help identify high-risk patients with both osseous and cartilaginous deficiencies who may benefit from earlier intervention. This approach mirrors recent recommendations for adolescent borderline dysplasia by Asturias et al. (41), where treatment decisions increasingly rely on a comprehensive assessment of symptoms and intra-articular morphology rather than radiographic angles alone. Thus, MRI should be considered when a mismatch between radiographic appearance and clinical function is observed.

This study has several limitations. First, the cross-sectional design of this study precludes the analysis of longitudinal changes. Future longitudinal studies are warranted to investigate the temporal evolution of anterolateral and lateral cartilaginous acetabular coverage of the femoral head after open reduction and its correlation with long-term acetabular remodeling. Second, this was a single-center, retrospective study with a small sample size, which may limit the representativeness of our cohort. The significant predominance of female patients introduces a potential for selection bias. Furthermore, the results may be influenced by confounding factors such as patient loss to follow-up or specific institutional admission criteria. Future multi-center collaborations are necessary to enhance the generalizability of our findings. Third, this study lacked a healthy control group. Future research should incorporate an age-matched healthy cohort to establish normative reference ranges for the A-CAHI and L-CAHI. Fourth, our analysis did not account for the potential influence of variables such as the pre-operative severity of dislocation or the post-operative immobilization protocol on functional outcomes. Finally, patient height and weight percentiles were not included in our analysis, and these anthropometric factors may represent potential confounders that could have influenced the results.

In conclusion, our study offers a new perspective on the assessment of DDH after open reduction. Our findings suggest that a multi-planar MRI assessment of cartilage may be a more reliable indicator of functional status than radiography. By offering a potential anatomical basis for the “imaging-function mismatch,” our work provides a rationale for considering the integration of MRI into the mid-term follow-up protocol. The thresholds identified in this study (A-CAHI ≥ 79.71 and L-CAHI ≥ 75.73) could serve as useful decision-support tools to help guide clinical management during this critical postoperative period.

## Data Availability

The original contributions presented in the study are included in the article/Supplementary Material, further inquiries can be directed to the corresponding author.
